# Global satellite-based sea and sea-ice surface temperatures since 1982

**DOI:** 10.1038/s41597-026-07363-4

**Published:** 2026-05-08

**Authors:** Pia Englyst, Ioanna Karagali, Ida L. Olsen, Guisella Gacitúa, Alex Hayward, Nishka Dasgupta, Johan H. Scheller, Jacob L. Høyer

**Affiliations:** Danish Meteorological Institute (DMI), National Center for Climate Research (NCKF), Sankt Kjelds Plads, Copenhagen Ø, 2100 Denmark

## Abstract

A 43-year Climate Data Record (CDR) of combined global sea and sea-ice surface temperature (SST/IST) from 1982 to 2024 has been produced from satellite observations within the Copernicus Climate Change Service (C3S). Infrared and microwave satellite data are integrated using optimal interpolation to provide daily, gap-free (L4, 0.05^°^) global SST/IST fields. Consistent and accurate sea-ice concentration (SIC) fields are derived by combining passive microwave SIC CDRs with sea ice charts, improving SST and IST characterization. The product also includes under-ice SST (UISST) derived from SIC and monthly salinity climatologies. Validation against in situ SSTs shows median differences of −0.04 ^°^C and robust standard deviations of 0.17–0.28 ^°^C. For sea ice, the median differences range from 1.41 ^°^C to − 4.62 ^°^C, with robust standard deviations of 2.55–4.99 ∘C. This CDR provides a novel and consistent dataset for assessing climate change and extremes in polar regions and globally, independently of sea-ice cover. From 1982–2024, global SST/IST increased by ~0.75 ^°^C, with amplified Arctic warming (~4.36 ^°^C) and modest Antarctic warming ~0.54 ^°^C), although recent years have been record-breakingly warm in Antarctica.

## Background & Summary

Sea Surface Temperature (SST) and sea-ice surface temperature (IST) are recognised as Essential Climate Variables (ECVs); observing them with high accuracy is crucial for monitoring, understanding and predicting changes in the climate system^1^. Traditionally, satellite-derived SST and IST products have been developed and analyzed separately, complicating assessments of surface temperature trends in high latitudes^2,3^, where the sea ice cover varies seasonally and inter-annually^4^. Most existing global gap-free (Level 4, L4) SST products provide an under-ice SST (UISST) estimate in sea-ice covered regions, representing the water temperature just below the sea ice^5–7^. UISST does not represent the surface temperature, as strong vertical gradients usually exist within snow and sea ice^8^, resulting in a weak coupling with the atmosphere. Consequently, using such products to determine high-latitude trends^9,10^ can lead to misinterpretation. A more consistent approach is to combine SST and IST estimates into a unified surface temperature product.

**Fig. 1:**
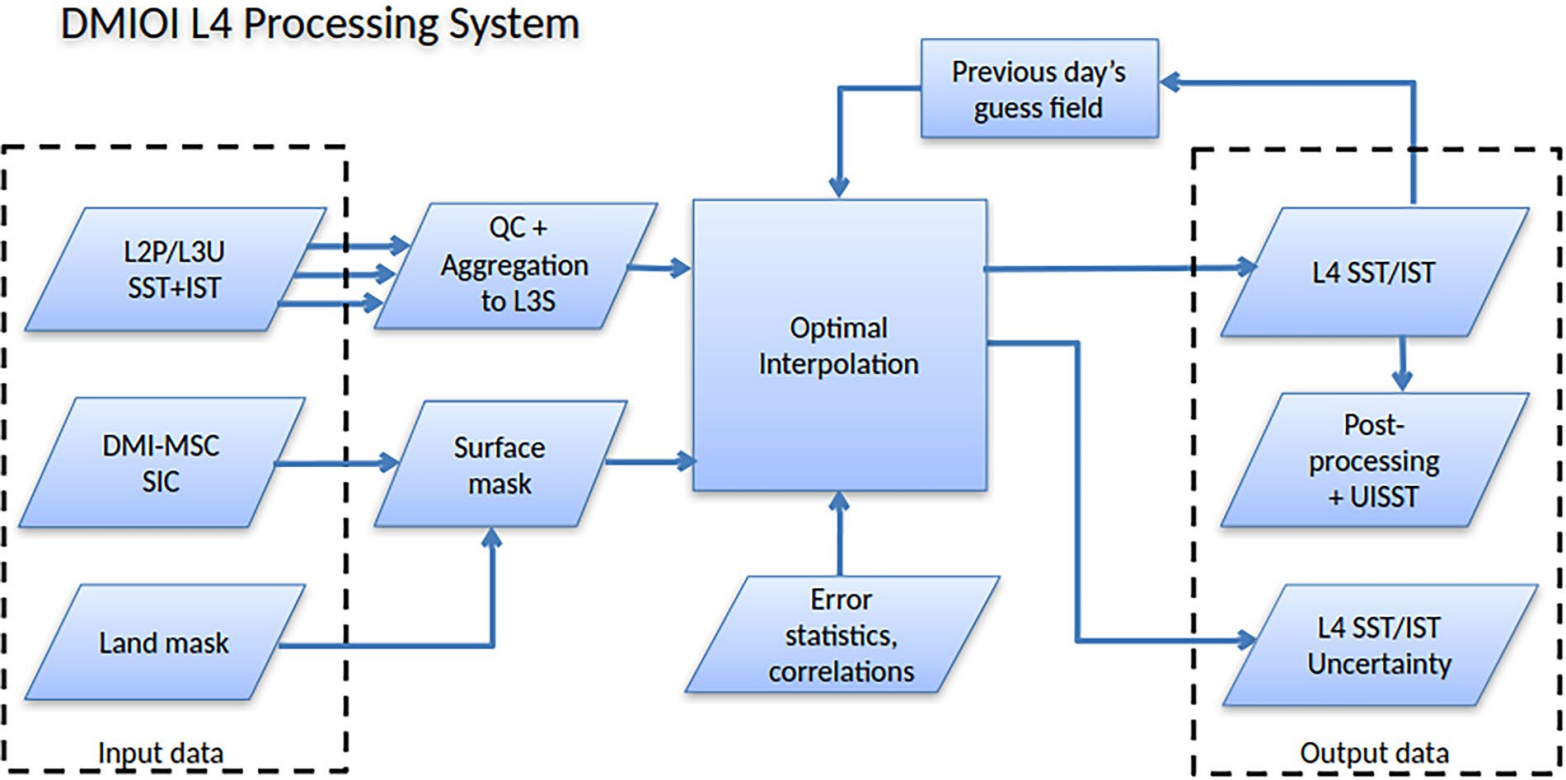
Schematic diagram illustrating all the processing steps of the DMI Optimal Interpolation (DMIOI) L4 Processing System.

The first L4 combined SST and IST Climate Data Record (CDR) enabled consistent surface temperature monitoring across open ocean, sea ice, and marginal ice zone in the Arctic^11^. Building on this effort, we present the first daily, global, satellite-derived, L4 CDR of combined SST and IST. By including the Southern Ocean and Antarctic sea-ice-covered waters, this dataset extends consistent surface temperature monitoring to regions which have experienced rapid recent changes^12–14^. This global L4 SST/IST CDR was developed within the Copernicus Climate Change Service (C3S) and covers the period 1982–2024 (with regular updates planned within C3S) at a spatial resolution of 0.05^°^. The dataset is generated using the Danish Meteorological Institute (DMI) L4 processing system based on Optimal Interpolation (OI)^11,15^, incorporating both infrared and microwave satellite observations. The input SST data include infrared Level 3 Uncollated (L3U) observations from the ESA Climate Change Initiative (ESA-CCI) (1982–2021)^16^ and C3S (2022–2023) as well as passive microwave Level 2 Processed (L2P) observations from ESA-CCI SST (2002–2017)^17^ with a subsequent temporal extension to 2024. The input IST data are obtained from L2P Arctic and Antarctic ice Surface Temperatures from thermal Infrared (AASTI) satellite CDR (1982–2019) and from C3S onwards^18^. Surface type classification is based on Sea Ice Concentration (SIC) information from the DMI Multi-Source Composite SIC (DMI MSC-SIC,^19^), which combines passive microwave SIC CDRs with sea ice charts and SST observations to produce accurate and consistent SIC fields since 1982. Figure 1 illustrates the main processing steps from the aggregation of the single sensor L3U and L2P observations into super-collated L3 (L3S) fields, which are then combined through OI to generate daily multi-sensor L4 SST/ISTs with corresponding uncertainties. UISST estimates in sea-ice covered regions are also provided for consistency with existing L4 SST products.

The C3S L4 SST/IST CDR is independent of in situ observations, enabling fully independent validation. SST validation against drifting buoys, Argo floats and tropical moored buoys shows median differences of −0.04 ^°^C and robust standard deviations of 0.17–0.28 ^°^C, consistent with results for ESA-CCI SST L4 CDR^16^. For Arctic sea ice, median differences relative to in situ near-surface air temperature range from −2.28 ^°^C to − 4.62 ^°^C, with robust standard deviations of 2.55–3.42 ^°^C, in agreement with earlier reportings^11,18^. Antarctic sea-ice validation results are comparable or slightly worse than those for the Arctic. However, the limited availability of in situ observations restricts both validation representativeness and long-term stability assessments.

The stability of the global SST component is −0.01 ^°^C/decade relative to drifting buoys, while Arctic IST stability is −0.08 ^°^C/decade based on available in situ measurements. Both meet the Global Climate Observing System (GCOS) “goal” requirements for long-term stability^1^, demonstrating the suitability of the dataset for long-term climate monitoring.

Between 1982–2024, global sea and sea-ice surface temperature increased by ~0.75 ^°^C, with significant stronger Arctic warming (4.36 ^°^C) and weaker Antarctic warming (0.54 ^°^C). However, recent years (2016–2024) have been exceptionally warm in the Antarctic region, consistent with record-low sea ice^12,20^. These recent developments may indicate the onset of stronger Antarctic warming, underscoring the importance of continued, consistent global surface temperature monitoring.

## Methods

This section describes the input data and processing scheme used to generate the C3S global L4 SST/IST CDR. Following the flowchart in Fig. 1, the section is structured to first describe the satellite input datasets, followed by the aggregation into L3S fields, the optimal interpolation (OI) process used to produce the combined multi-sensor daily L4 SST/IST fields and associated uncertainties, and finally the post-processing steps, including the addition of UISST estimates and corresponding uncertainties in sea-ice-covered regions.

### Input Data

**Fig. 2:**
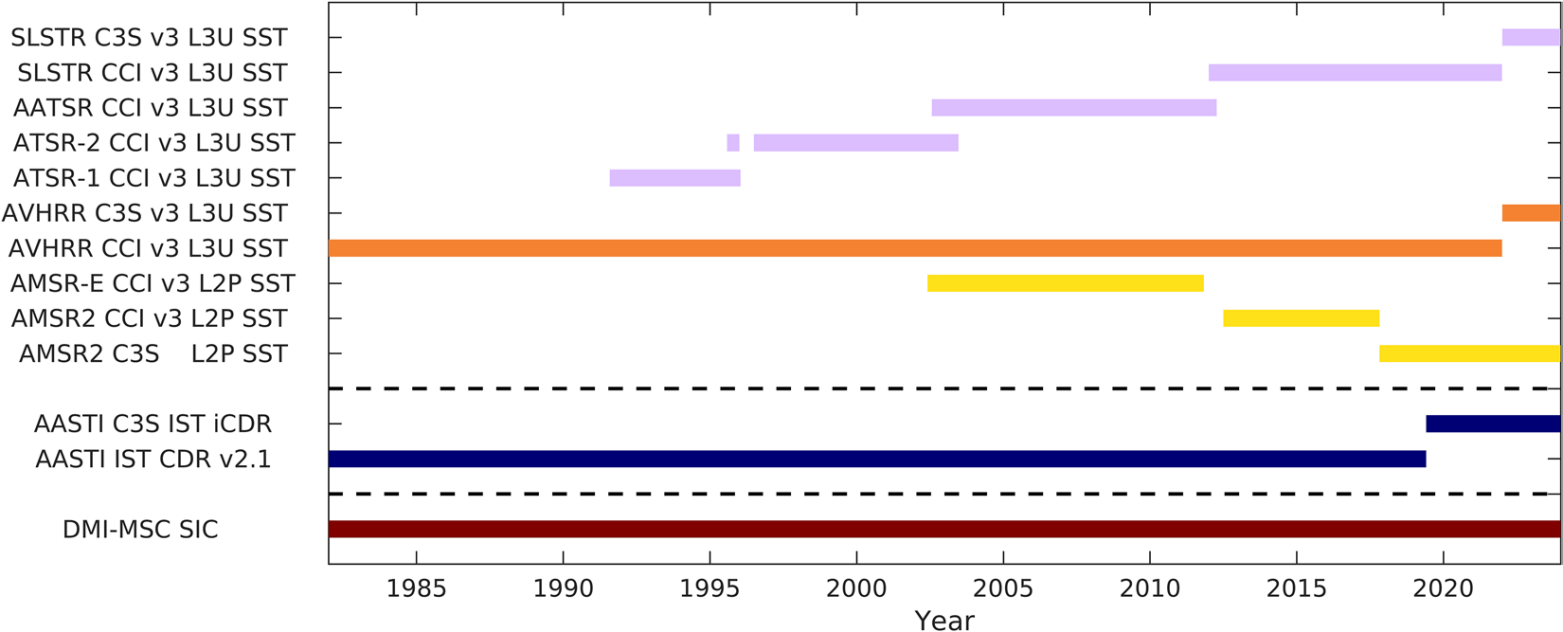
Temporal coverage of the DMI-MSC SIC product and the L2p and L3U IST and SST satellite data used as input to the DMI OI Processing System.

The C3S global L4 SST/IST CDR is based on several sources of satellite observations. Figure 2 provides a full overview of the different datasets used in the production and their respective temporal coverage. As input for SST, the L3U SST CCI CDR version 3.0^16^ is used for the period 1982–2021. The SST CCI CDR includes observations from the Along Track Scanning Radiometer (ATSR) 1 instrument on board the European Research Satellite (ERS-1) platform, ATSR 2 on board the ERS-2 satellite, the Advanced ATSR (AATSR) on board Environmental Satellite (ENVISAT)^21^, the SLSTR A/B instruments on board the Sentinel 3 satellites^22^ and the Advanced Very High Resolution Radiometer (AVHRR) onboard the National Oceanic and Atmospheric Administration (NOAA) and Meteorological Operations (MetOp) satellites^23^. The CDR is combined with an extension (based on the same software and systems) from 2022–2025 provided as an Interim CDR (ICDR)^24^ funded by C3S, the UK Earth Observation Climate Information Service (EOCIS) and UK Marine and Climate Advisory Service (UKMCAS). The ICDR includes infrared observations from the SLSTR A/B instruments on board the Sentinel 3 satellites and the AVHRRs on board the NOAA and Metop satellites. The SST CCI CDR also includes passive microwave (PMW) SSTs from the AMSR-E and AMSR2 sensors for the period June 2002-October 2017^17,25^. After this period, a consistent extension has been run by DMI and University of Reading based on the same retrieval algorithm and processor developed within CCI. For both the infrared and microwave products, the “SST depth” variable is used, which is adjusted to represent a depth of 20 cm (comparable to in situ drifting buoy measurements) at the 10:30 or 22:30 local time of day (when the diurnal cycle in SST is usually near its daily average) to best represent the daily average^16,26^.

In sea-ice covered areas, L2P observations are included from the Arctic and Antarctic ice Surface Temperatures from thermal Infrared satellite sensors (AASTI) climate dataset version 2 (1982–2014), produced by DMI and MET Norway, and its Interim climate dataset extension from 2019 onwards, produced within C3S, based on the series of AVHRR sensors on board the NOAA and Metop satellites^18,27^.

In the production of the C3S global L4 SST/IST CDR, sea ice information is needed to identify the open water, marginal ice zone and sea-ice covered regions. In this case, the DMI Multi-Source-Composite Sea Ice Concentration (DMI-MSC-SIC^19^) is used. The DMI-MSC-SIC is based on existing satellite passive microwave CDRs (OSI-458, SICCI-HR-SIC and OSI-450-a) and sea ice charts (USNIC, CMEMS/FMI/SMHI) with the aim to use the best available data at all times in all regions. The passive microwave based CDRs are used as the primary input and sea ice charts are used to provide landfast ice (USNIC), for filtering (USNIC) and in the Baltic Sea (CMEMS/FMI/SMHI). To ensure a consistent data product several filters, including one using the USNIC ice charts, and a bias adjustment have been applied^19^.

### L3S aggregation

The input L2P and L3U SST and IST observations have been aggregated into daily L3 single sensor fields with a 0.05 degrees resolution by averaging all available observations (meeting the minimum quality level) within a given aggregation window. The length of the aggregation window varies throughout the record due to the varying number of available satellite observations. Based on the number of L3U files per year, a fixed temporal aggregation window of ±24 h was used for most of the record. However, from the period 1982–1986, when limited input observations were available, the window was extended to ±60 h. A minimum Quality Level (QL) of 4 was used for all satellite observations except from those from AMSR, which were assigned with a minimum QL of 3. This choice reflects a trade-off between data quality and spatial coverage. For AMSR SST, higher QLs are defined based on retrieval uncertainty^17^, and regions where PMW observations provide the greatest benefit (such as the Arctic) often exhibit elevated uncertainties^17,28^. Restricting inputs to QL ≥4 would therefore reduce coverage and introduce a temperature- and latitude dependence in the sampling, given the inherent uncertainty characteristics of PMW retrievals (i.e. elevated uncertainties in cold waters)^17,28^. To avoid this, QL 3-5 observations are included, while the increased uncertainty is mitigated through the L3 aggregation and OI, which statistically accounts for the single-sensor uncertainties in the final product. In high-latitudes (i.e. colder waters), where QL 3 data are more prevalent, the higher revisit frequency of the polar orbiting satellites further reduce the aggregated uncertainty. Finally, previous work has shown that including QL 3-5 PMW SST observations improves L4 SST estimates in the Arctic^29^. A constant clear-sky bias correction of 0.85 ^°^C has been applied to the derived AASTI/C3S L3 fields based on previous findings^30^.

The single sensor L3 fields were afterwards aggregated into daily L3S fields by calculating the noise weighted average of all available L3 fields. Based on L3 validation results against in situ observations, we have assumed uncertainties of 0.3 ^°^C, 0.4 ^°^C and 0.5^°^C for ATSR, SLSTR and AMSR, respectively. The AVHRR uncertainties depend on the year, with assumed uncertainties of 0.6 ^°^C before 1989, 0.5 ^°^C between 1989–2004, and 0.4 ^°^C for years later than 2004. The L2P AASTI/C3S IST uncertainties are assumed to include a seasonal component, with maximum uncertainties of 1.3 ^°^C in December and a minimum uncertainty of 0.8 ^°^C in June. This seasonality is attributed to differences in the cloud-screening performance between dark and sunlit periods^31^. In addition, a second seasonally varying uncertainty is assumed to be introduced during the L3 aggregation of AASTI observations due to sampling effects. This component is also assumed to peak in December (0.7 ^°^C) and reach a minimum in June (0.2 ^°^C), reflecting the greater temporal variability of surface temperatures in winter compared to summer^30,32,33^.

Finally, before entering the OI processing system, any systematic inter-sensor biases must be corrected to ensure consistency across the multiple satellite sensors used over the 43-year period, as uncorrected changes in sensor characteristics could introduce artificial trends and undermine confidence in the long-term record. This inter sensor harmonization, particularly for the IR sensor series, has already been addressed in the ESA CCI SST project (on which our SST data are based) through documented harmonization and cross sensor alignment procedures^16,34^. To adjust the passive microwave SSTs, the L3 SST fields from AMSR-E and AMSR2 were bias-corrected against the IR (AVHRR) SSTs using a dynamic inter-sensor bias correction scheme developed specific for high latitudes^35^. For each day, an IR SST reference field was aggregated to a 0.25^°^ grid within a 7-day window, and the daily differences between the IR reference field and the corresponding coarse resolution aggregated PMW field were calculated, interpolated to 0.05^°^, and smoothed over 500 km to reduce small-scale noise while capturing slowly varying systematic biases. The bias correction scheme has previously been applied to combine IR and PMW SSTs in the Arctic, where it efficiently removed inter-sensor biases throughout the year, providing a robust basis for merging IR and PMW sensors into a consistent long-term SST record^29^.

### Optimal interpolation

The DMI OI L4 processing framework (Fig. 1) is applied here using the same approach as in previous studies^11,15,35^; for a detailed description of the OI methodology, including equations and implementation details, the reader is referred to these works. The daily L4 fields are produced by performing a statistical OI, which provides the best SST/IST estimate in each grid cell based on the aggregated L3S fields and their associated uncertainties, a first-guess field, a surface mask and a number of OI statistical parameters (described below). The OI method also provides an uncertainty estimate for each grid cell, which depends on the data availability, the background field, the uncertainty of the observations and the proximity of the observations to the estimation point.

The OI is performed on anomalies, and uses the previous day’s analysis as the first-guess field. This approach ensures that the first guess estimate is preserved in regions with sparse or missing observations. The surface mask combines a static land mask from the SST CCI v3^16,36^, used for land–ocean classification, with a daily varying sea-ice mask derived from the DMI-MSC SIC product^19^. The SIC field is used to differentiate between open ocean (SIC < 15%), marginal ice zone (MIZ; SIC 15–70%) and sea ice (SIC > 70%). All rivers and lakes, except the Caspian Sea, are masked out and are thus not covered by the product. The surface mask is used within the OI scheme to identify the surface type and ensure that only appropriate observations contribute to each estimation point. Specifically, AASTI/C3S observations over sea ice and MIZ are used for estimation points classified as sea ice, while CCI/C3S SST observations from open water and the MIZ are used for open-water estimation points. In the MIZ, the following observations are used: AASTI/C3S MIZT (SIC 15–70%), IST (SIC > 70%), and CCI/C3S SST (SIC < 15%). This means that the MIZ integrates observations representative of both open ocean and sea-ice-covered conditions. Within the OI analysis framework, the MIZ is treated as a distinct surface type with its own set of statistical parameters designed to capture its unique variability and spatial structure.

The required OI statistical parameters are the first guess variances and correlation functions. These have been derived separately for open ocean, sea ice and the MIZ, using the DMI-MSC SIC to identify the surface type. The first guess error variances were derived from eight years (1982–1983, 1992–1993, 2002–2003, and 2012–2013) of L3 observations, where each day was compared to the previous day’s analysis and spatial first guess variances were then calculated from the anomalies. In this case, monthly mean (and spatially varying) first guess variances were used to capture the seasonal variability, which is particularly large in sea-ice covered regions. Spatially varying correlation functions in the latitudinal and longitudinal directions were derived empirically from the observations (i.e. one year (2015) of anomalies) under the assumption of steady state^11^. Separate correlation functions were obtained for SST, IST and the MIZ, to account for the differing characteristics of these surface types, but all functions share the same form. The correlations were calculated by correlating time series of anomalies within bins of 10 × 10^°^ for SST, 50 × 50^°^ for IST, and 15 × 15^°^ for the MIZ. Random noise was estimated and removed for each surface type prior to fitting the correlation functions. The resulting correlation functions are assumed to be constant in time.

**Fig. 3:**
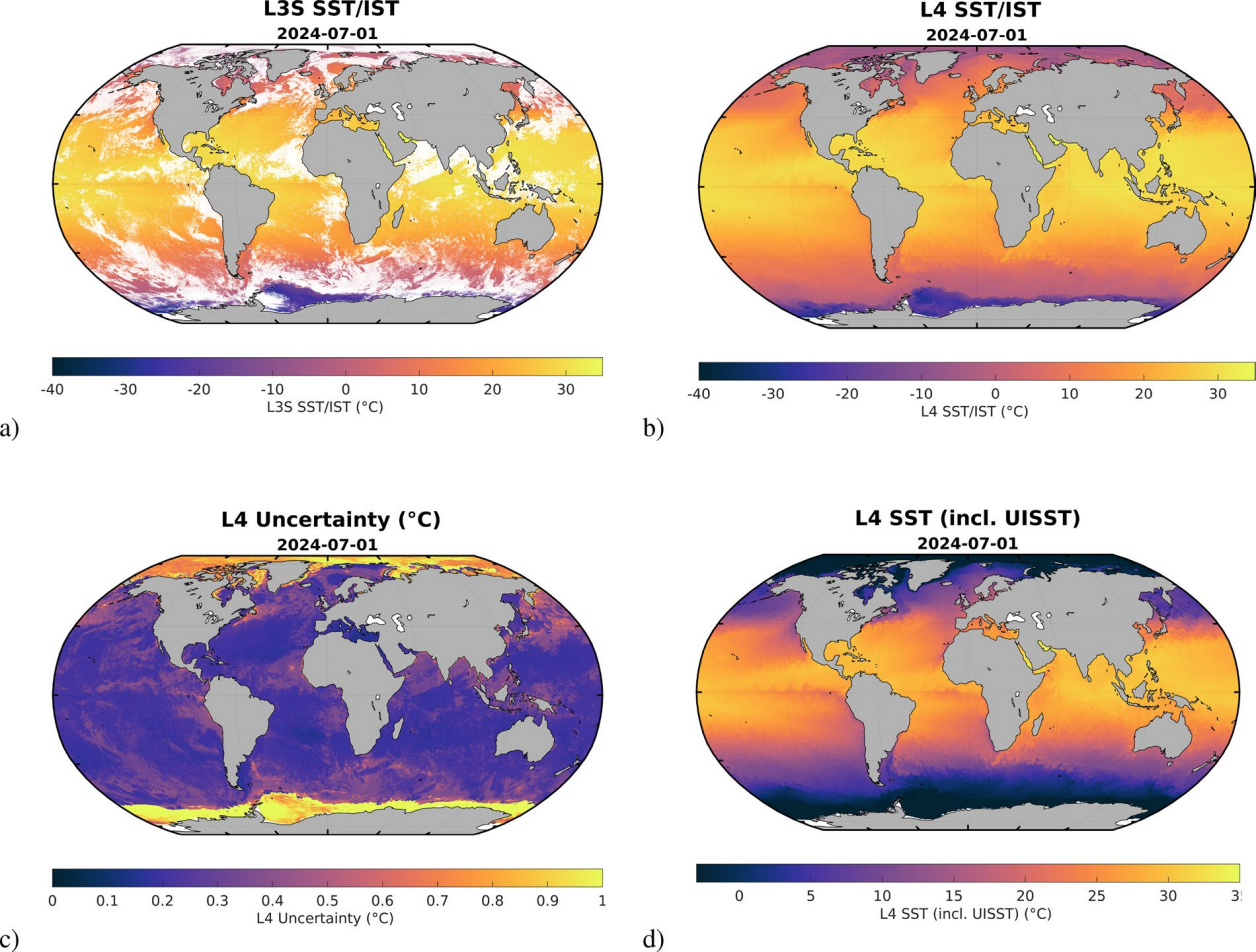
Examples illustrating the **a**) L3S SST/IST field, **b**) L4 SST/IST field, **c**) L4 Uncertainty field, **d**) L4 SST field including under-ice SST (UISST) in sea ice covered regions for July 1, 2024.

For each estimation point, correlations were computed for all observations within a 100 km radius. From each quadrant surrounding the estimation point, the observation with the highest correlation was selected (if available). This process was repeated until either the maximum number of observations (set to 8) was reached or no additional observations were available. The resulting correlations, along with first guess variances and satellite uncertainties, were then used to assign appropriate weights to each observation in the OI scheme in order to produce the gap-free surface temperatures fields and corresponding uncertainties. Figure 3 presents an example from July 1, 2024 showing the global L3S SST/IST field, the L4 SST/IST field and its corresponding uncertainty field, and the L4 SST field including UISST in sea-ice covered regions.

### Post-processing

A number of postprocessing steps have been applied to ensure consistency, completeness, and quality of the final dataset. The first step addresses pre-existing inconsistencies between the combined SST/IST product and the underlying SIC product. In some cases, the initial fields exhibit inconsistencies between the ice mask and the surface temperatures, where pixels with temperatures below −1. 8 ^°^C were classified as open water. These inconsistencies can arise for several reasons including retrieval limitations, mismatches in the temporal and spatial scales, and the interpolation of MIZ (15–70% SIC) observations into adjacent open-water or sea-ice-covered regions. Since, the SIC product is derived from relatively coarse-resolution satellite sensors, whereas the SST/IST fields are based on higher resolution infrared observations, the surface temperatures are assumed to better represent the true local conditions. Consequently, a SIC adjustment is applied during the post-processing: in grid cells where SIC is zero but the assigned IST is below the freezing point, the SIC value is extrapolated from neighboring ice pixels using a nearest neighbor approach. All extrapolated values are flagged by introducing an additional variable, *sea_ice_fraction_flag*, which is binary (0 = original SIC, 1 = extrapolated SIC). Using this flag, the original SIC field can be restored by replacing extrapolated SIC values with SIC = 0%.

A second post-processing step were applied to reduce noise and small-scale artifacts in the IST field, which may arise from contamination by undetected clouds. A binary mask is defined for grid cells with non-zero sea-ice concentration, and a Gaussian filter (*σ* = 2 grid cells) is applied to both the masked IST field and the mask itself. This procedure yields a locally averaged IST field in which only neighboring ice pixels contribute to the smoothing. The smoothed values replace the original ISTs over sea ice, while land and open-water pixels remain unchanged, preserving physical boundaries.

### Under ice sea surface temperature (UISST)

To ensure consistency with the existing global L4 SST products, such as the CCI SST L4 CDR, two additional variables are included over ice-covered regions: under-ice SST (UISST) and its associated uncertainty. UISST is computed using a methodology where SIC and the climatological freezing-point are combined into a proxy for the SST under ice^5^.

1 $$UISST={T}_{f}+C(1-SIC).$$

**Fig. 4:**
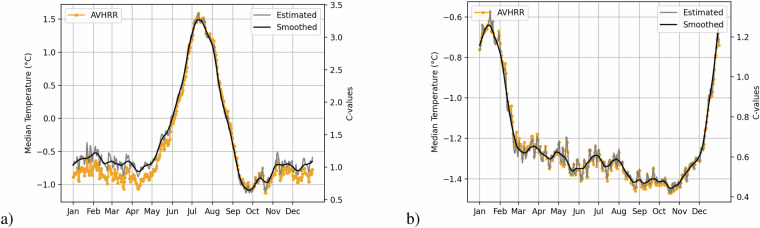
Seasonal cycles of C-values and AVHRR temperatures in **a**) the Northern Hemisphere and **b**) the Southern Hemisphere. Orange lines show AVHRR temperatures (^°^C) plotted on the left y-axis. Grey lines show estimated daily C-values and black lines show LOESS-smoothed C-values, both plotted on the right y-axis.

The freezing temperature (*T*_*f*_) is computed as a function of salinity (*S*) and surface pressure (*p*)^37^ using monthly salinity fields from the World Ocean Atlas 2023 (WOA23, 1991–2020 climatology^38^) are used as input. UISST is parameterized as a transition between open-ocean SST and the local freezing temperature, with increasing SIC, constraining the surface temperature towards *T*_*f*_. In equation (1), the coefficient *C* controls the sensitivity of UISST to SIC. *C* is derived from AVHRR CCI SST L3 observations (1992–2011) in low-ice regions (0 < SIC < 15%), to ensure that the impact from ice is minimized. Estimated daily *C* values are derived separately for each hemisphere and then smoothed using non-robust locally weighted scatterplot smoothing (LOWESS) with frac = 0.05 to suppress day-to-day noise and preserve the seasonal cycle (Fig. 4).

The associated UISST uncertainty is defined as:

2 $$\Delta UISST=\sqrt{{(\Delta {T}_{f})}^{2}+{(C\cdot \Delta {\rm{SIC}})}^{2}},$$

where *ΔT*_*f*_ is the uncertainty in the freezing temperature and *Δ*SIC is the uncertainty in sea-ice concentration. Uncertainty in *T*_*f*_ is estimated by propagating the salinity uncertainty through the salinity dependence of *T*_*f*_, assuming constant *p*: $\Delta {T}_{f}=|\frac{\partial {T}_{f}}{\partial S}|\Delta S$. Here *ΔS* is taken from WOA23 standard error of analysis, defined as the standard error about the statistical mean of practical salinity in each grid cell and depth level. To obtain a global annual proxy suitable for UISST error propagation, the WOA23 salinity uncertainty field was weighted by observation density and conservatively rescaled to 0.33, corresponding to the 99th percentile of standard error values to account for sparse polar observations. This scaling is consistent with reported salinity climatology uncertainty of 0.3 or larger in the upper 50 m of coastal ocean waters^39^. SIC uncertainty is obtained using the product uncertainties from one year of PMW SIC CDRs (OSI-458, SICCI-HR-SIC and OSI-450-a) that were used as input to the DMI-MSC. For each 1% SIC bin, the average of the product uncertainties in the SIC products is used, with the largest values occurring for intermediate ice concentrations (0.15−0.6). Salinity uncertainty contributes only modestly to *ΔT*_*f*_ and Eq. (2) shows that UISST uncertainty is primarily driven by both *C* and *ΔSIC*. This effect is most pronounced in the Arctic during May-August, when *C* can exceed 3.3, causing propagated uncertainties to approach 1 ^°^C in the MIZ. From September to April, *C* remains near 1, resulting in lower UISST uncertainties of about 0.3 °C.

## Data Records

The Global L4 SST/IST CDR is available from the Copernicus Climate Change Service (C3S) Climate Data Store (CDS)^40^ and distributed under the Creative Commons Attribution 4.0 International license (CC-BY 4.0; https://creativecommons.org/licenses/by/4.0/). The files are provided in netCDF-4 classic format following: Climate-Forecast (CF) metadata conventions^41^ and the GHRSST Data Specifications^42^. The files are supplied on a regular 0.05^°^ latitude/longitude grid (based on the WGS1984 geoid) and the variables have dimensions 3600 × 7200, corresponding to the number of grid cells in the latitude and longitude direction, respectively. The key variables present in the files are listed in Table 1. It should be noted that the “lake” identifier in the product mask exclusively refers to the Caspian Sea. All other lakes are not represented and are thus classified as land within the dataset.

**Table 1:** Key data variables included in the C3S global L4 SST/IST CDR.

Name	Units	Description
analysed_st	K	Daily mean estimate of surface temperature (ST; consisting of SST, IST and MIZT).
analysed_sst	K	Daily mean estimate of sea surface temperature (SST) including under-ice SST (UISST) in ice covered regions.
analysis_error_st	K	Estimated standard error (uncertainty) in analysed ST.
analysis_error_sst	K	Estimated standard error (uncertainty) in analysed SST.
sea_ice_fraction	—	Sea ice fraction, ranging from 0 to 1.
sea_ice_fraction_flag	—	Flag indicating where the sea_ice_fraction is the original field (zero) and where it has been extrapolated (one).
mask	—	water, land, optional_lake_surface, sea_ice, optional_river_surface for 1B, 2B, 4B, 8B, 16B

## Technical Validation

This section presents the analyses that are needed to support the technical quality of the C3S global L4 SST/IST CDR. This includes a validation of the SST and IST components against independent in situ observations (including a stability assessment) and an evaluation of the uncertainty estimates. A climate assessment has also been performed to highlight the trends and variabilities observed in the C3S global L4 SST/IST CDR over the full record (1982–2024).

### SST validation

The in situ SST measurements used for validation are extracted from the Met Office Hadley Centre Integrated Ocean Dataset (HadIOD) v1.2.0.0^43^. The reference data are referred to as the SST CCI Independent Reference Data Set (SIRDS; https://www.metoffice.gov.uk/hadobs/hadiod/sirds.html) and have previously been used to validate the SST CCI products^16,17,44^. The SIRDS include measurements from various platforms of which we will use the drifting buoys, Global Tropical Moored Buoy Array (GTMBA), ships, and Argo floats. The various data types have different characteristics resulting from varying instrument types, measurement uncertainties, sampling frequencies, and temporal and spatial coverage. These are described and discussed in great detail in^44^.

Daily averaged in situ measurements have been matched to the corresponding daily C3S L4 SST (SIC < 15%) whenever a daily averaged in situ value is available within a grid cell. Table 2 summarizes the validation results for each in situ type using robust statistics, specifically the median and robust standard deviation (RSD, 1.4826 times the median absolute deviation). The varying characteristics of the in situ types are evident, with the largest median difference (−0.12 ^°^C) and RSD (0.87 ^°^C) for ships, which is mainly explained by the larger uncertainties typically associated with ship-based measurements^45^. Drifting buoys and Argo floats have similar statistics with a median difference of −0.04^°^C and RSDs of 0.28 ^°^C and 0.23 ^°^C, respectively. The tropical regions are represented through the GTMBA, with median differences of −0.04 ^°^C and RSD of 0.17 ^°^C.

**Table 2:** Summary of robust validation statistics for the C3S global L4 SST (SIC < 15%) component against reference in situ SST observations.

Type	MD (^°^C)	RSD (^°^C)	Stability (^°^C/decade)	NUM	Period
Drifting Buoys	−0.04	0.28	−0.010	77,580,459	1982–2024
Argo	−0.04	0.23	−0.022	1,992,549	2000–2024
Ships	−0.12	0.87	−0.023	33,631,294	1982–2024
Moorings (GTMBA)	−0.04	0.17	0.002	255,094	1998–2024

The validation statistics include the Median Difference (MD) and the Robust Standard Deviation (RSD) of the differences. Stability refers to the slope of a linear trend in the MD (^°^C per decade). NUM refers to the number of daily L4 SST matchups with in situ observations, and period refers to the period within the matchups are available.

**Fig. 5:**
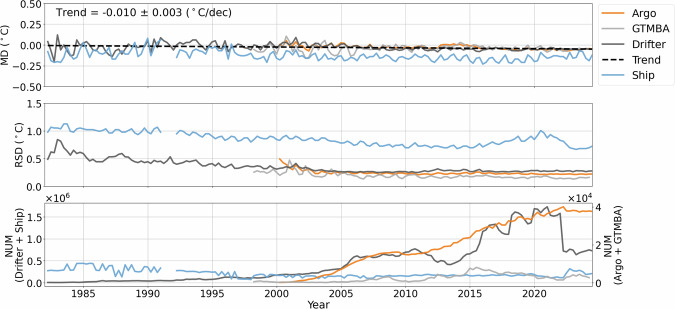
Timeseries of the C3S global L4 SST (SIC < 15%) validation results against in situ observations in terms of seasonal median differences (MD) and a corresponding linear trend for drifters (upper panel), Robust Standard Deviations (RSD) of the differences (middle panel) and the number of matchups (NUM, lower panel). The seasonal statistics are only calculated if > 45 matchups are available.

The validation against in situ observations generally improves through the timeseries (Fig. 5), reflecting improvements both in the in situ network and in the C3S global SST/IST product (mainly caused by improved satellite instruments and better satellite coverage over time). Around 2002, there is a noticeable improvement in the validation statistics and this is attributed to the introduction of new satellite sensors^16,44^. Ship observations are more uncertain and less stable (e.g. with a pronounced seasonal effect) than the other in situ types, but they are the main source of observations until the 2000s when the number of Argo and drifter observations increase greatly. The dashed line represents the trend in the median difference against drifting buoys, with a stability measure of −0.01 ^°^C / decade, which is equal to the GCOS “goal” stability for SST^1^. This agreement with drifting buoy observations provides an independent assessment of long-term stability and indicates that harmonization across multiple satellite sensors within the SST CCI project, together with the dynamic IR-PMW inter-sensor bias correction applied here, effectively minimizes residual biases and prevents artificial trends, thereby strengthening confidence in the robustness of the derived long-term trends.

**Fig. 6:**
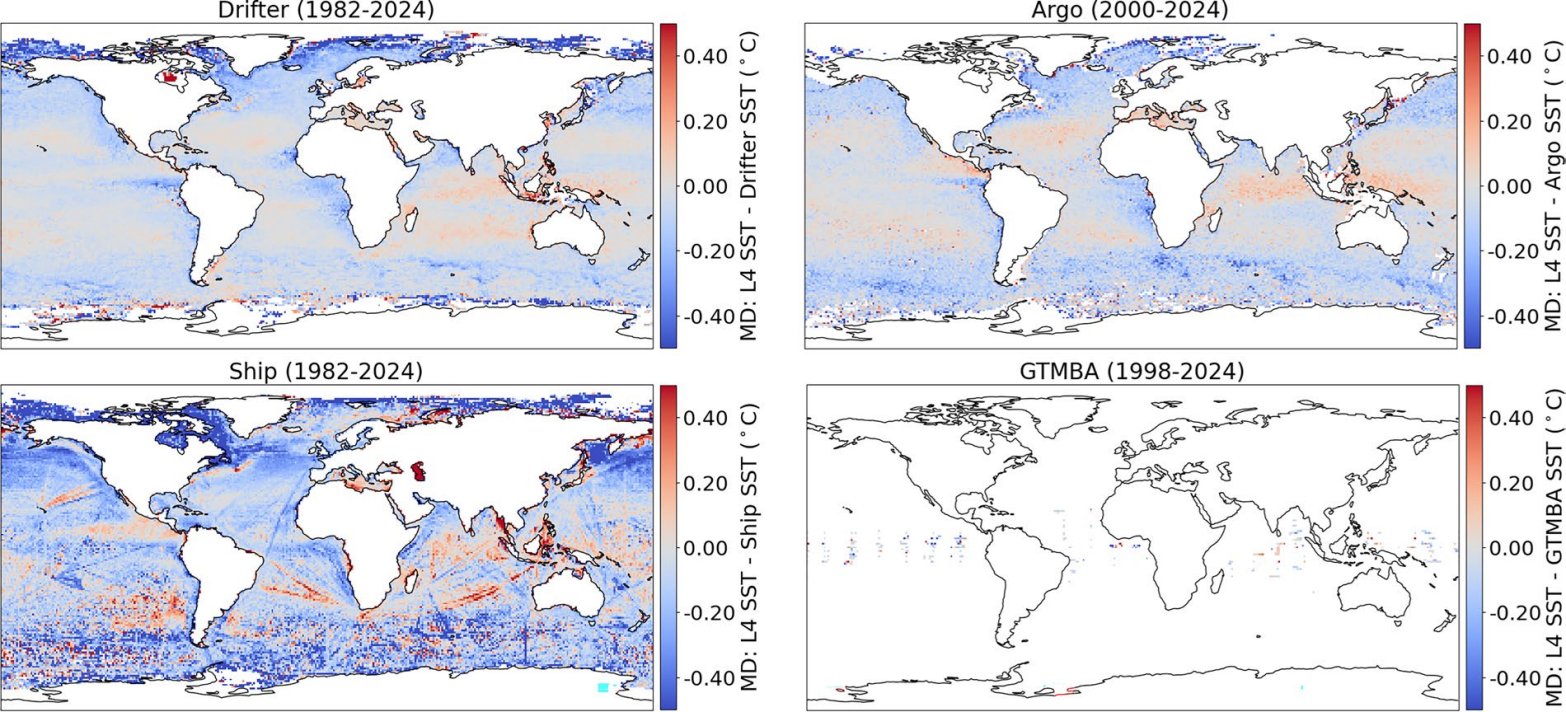
Spatial distribution of Median Differences (MD) between C3S L4 SST (SIC < 15%) and drifters, Argo floats, GTMBA and Ships, respectively. The time spans denote the period for which observations are available for the given in situ type. The statistics are calculated for each 1 × 1 degree grid.

**Fig. 7:**
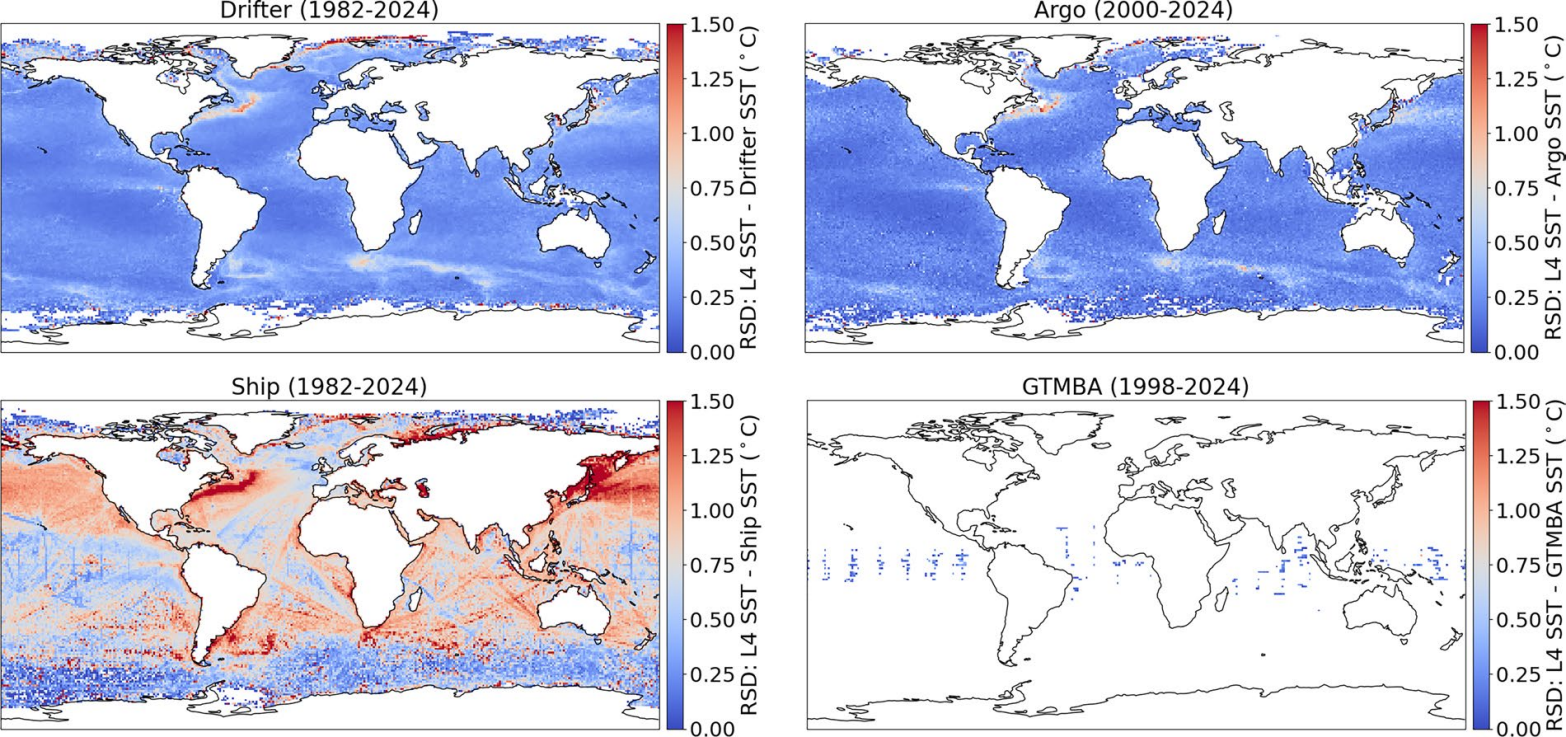
Spatial distribution of Robust Standard Deviations (RSD) between C3S L4 SST (SIC < 15%) and drifters, Argo floats, GTMBA and Ships, respectively. The time spans denote the period for which observations are available for the given in situ type. The statistics are calculated for each 1 × 1 degree grid.

**Fig. 8:**
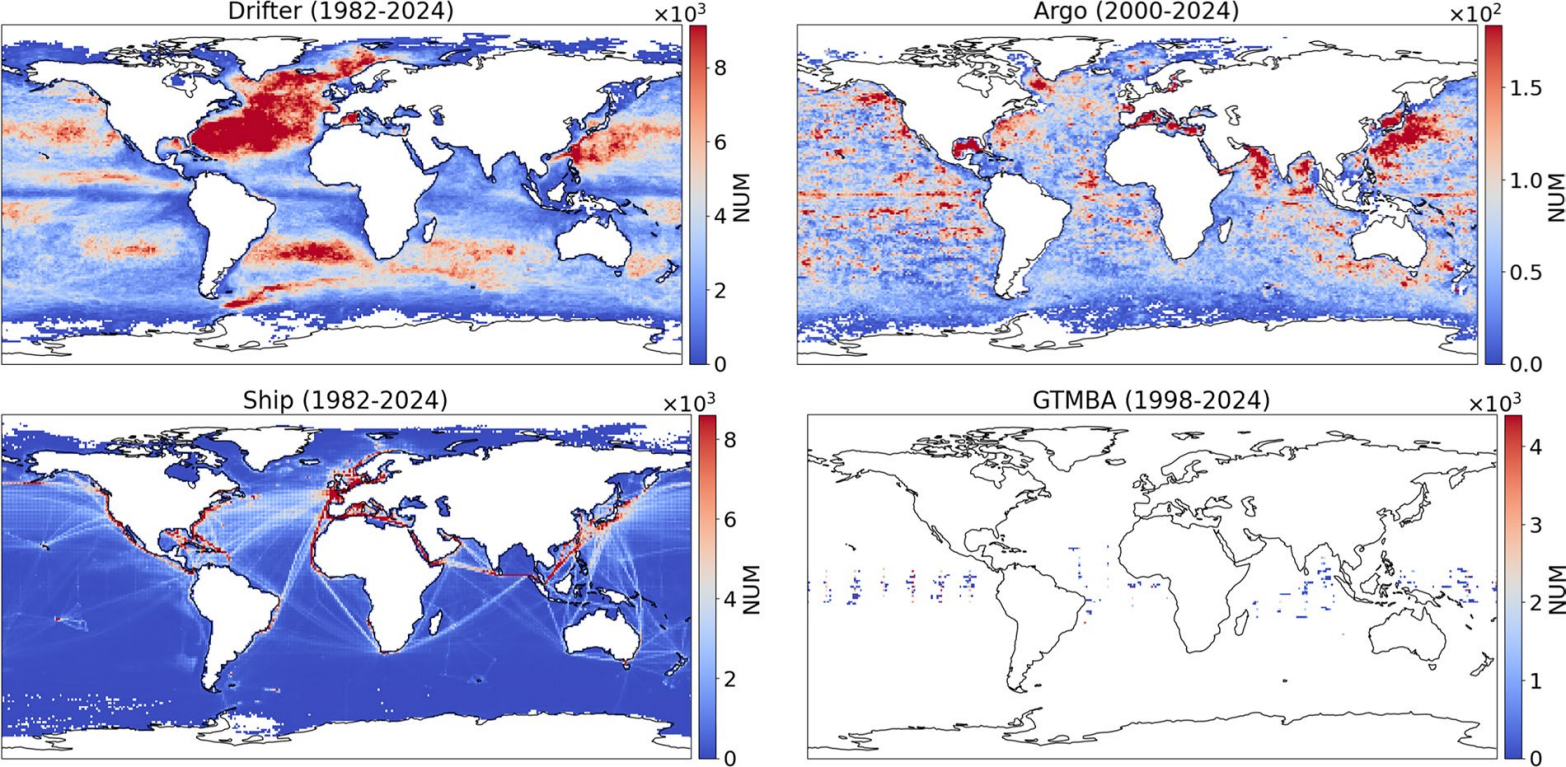
Spatial distribution of the total number of matchups (NUM), available within each grid cell, with drifters, Argo floats, GTMBA and Ships, respectively. The time spans denote the period for which observations are available for the given in situ type. The statistics are calculated for each 1 × 1 degree grid.

**Fig. 9:**
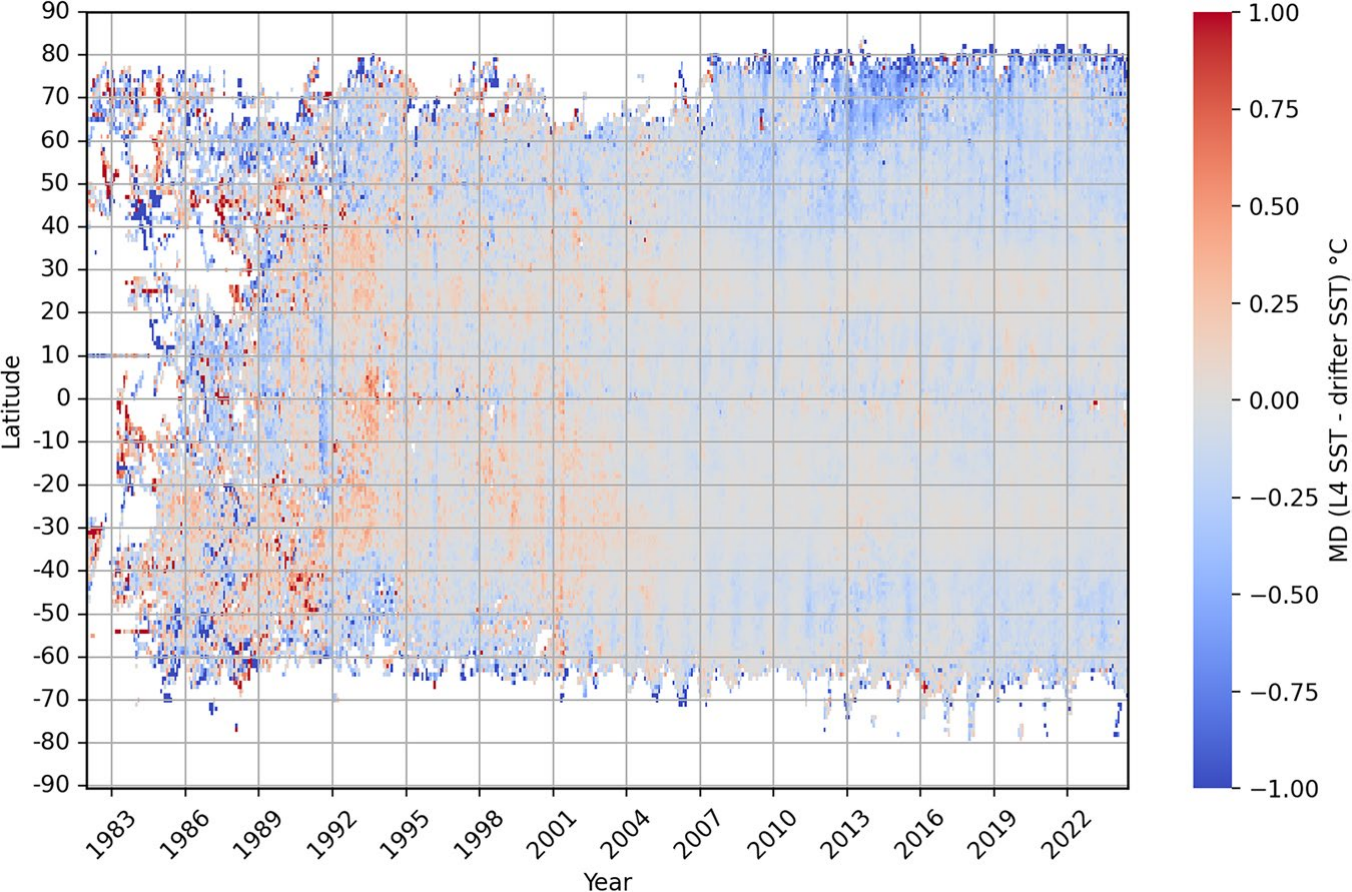
Hovmöller distribution of median SST difference between the C3S global L4 SST (SIC < 15%) and drifter observations from 1982 to 2024.

Figures 6, 7, 8 show the spatial distribution of the median difference, robust standard deviation, and the number of matchups between the C3S L4 SST and drifters, Argo floats, ships, and moored buoys (GTMBA). Generally, smaller median differences are observed in the mid latitudes, while the largest differences are observed in the Arctic. The largest RSDs are, as expected, observed in areas with large variability and strong SST gradients, such as along the Gulf Stream. Furthermore, higher and more variable RSDs are seen in higher latitudes, particular for the northern hemisphere and in areas close to the sea ice edge (e.g. the Greenland Sea and Barents Sea). An overview of the spatial performance for the complete time series is provided in Fig. 9 where the Hovmöller distribution of the monthly median difference between C3S L4 SST and drifter SST is plotted as a function of latitude. The largest median difference are seen in the early years, when only one or two AVHRR sensors were available^16^, and in the Arctic where the C3S L4 SST is colder than drifters, in particular in the years 2012–2016. The validation results presented in this section are in close agreement with those reported for the ESA CCI SST version 3^16,44^, indicating that they primarily reflect the performance of the input satellite data.

### IST validation

Validation of the L4 IST (SIC > 15%) product is constrained by the limited availability of in situ observations and by the elevated uncertainties associated with in situ measurements in ice-covered regions compared to open ocean conditions. Direct observations of the sea-ice surface temperature are extremely limited in both hemispheres. As a result, near-surface air temperatures are often used for the development and validation of IST retrievals and products^11,18^, despite known differences between the two variables^30^. This is also the case here, where the majority of the IST validation is based on near-surface air temperature observations from multiple sources, which combined span multiple decades. These include observations from ice drifting buoys obtained from the Meteorological Archival and Retrieval System (MARS) at the European Centre for Medium-Range Weather Forecasts (ECMWF), covering the period 1993–2014 (hereafter referred to as ECMWF buoys). For the northern hemisphere these are supplemented with data from U.S. Army Cold Regions Research and Engineering Laboratory (CRREL) mass balance buoys from 2001–2017^46^ as well as observations from the Cryosphere Innovation Seasonal Ice Mass Balance Buoys 3 (SIMB3) covering the period 2019–2024^47,48^ and from Russian North Pole (NP) drifting ice stations, mainly covering the period 1982–1989, with additional observations from 2003–2012^49^. These datasets have undergone manual quality control to identify and remove data artifacts and they have previously been used for validation of L3 and L4 ISTs^11,18^. In addition, observations from snow buoys (SB) and automatic weather stations (AWSs) were downloaded in July 2025 from the AWI Meereisportal^50^. In total, 42 snow buoys and three AWS were utilized covering the period from February 2013-December 2024 and January 2016-March 2017, respectively. Snow buoys recorded air temperatures at a reported 1̃.5 m height, while the AWS measurements were recorded at an estimated 1̃.75 m height from the surface. These data went through an automatic quality control workflow consisting of multiple filtering steps applied to temperature and position data from each buoy. Observations were included in the IST validation if all quality checks were passed. Finally, both IST and 2-meter air temperatures were included from the Multidisciplinary drifting Observatory for the Study of Arctic Climate (MOSAiC) expedition of the research vessel Polarstern in the Arctic Ocean from October 2019 to September 2020^51^. Temperature recordings are available from four measurement sites during the MOSAiC expedition: the Met City main site and three Atmospheric Surface Flux Stations placed around the Met City main site (ASFS30, ASFS40, and ASFS50), although none of the stations were running continuously throughout the MOSAiC period^52^. The MOSAiC data were downloaded from the NSF Arctic Data Center and include quality flags, and only the temperature observations with a quality flag of 0 (indicating high quality data) were used in the validation.

The near-surface air temperature measurements from ECMWF, CRREL, SIMB3, NP, AWI and MOSAiC are taken at varying heights, generally around 1.5–2 meters, but subject to changes due to snow accumulation, snow drift, and melt. For simplicity, these measurements will all be referred to as T2m measurements in the following. In addition to the T2m data, we use sea-ice surface temperature observations from 153 NASA IceBridge (IAKST1B) flights conducted between 2012 and 2019, primarily during March-May (version 2)^53^. These measurements are derived from infrared radiation data collected by a Heitronics KT-19 pyrometer, assuming a constant emissivity of 0.97. Originally provided at a spatial resolution of approximately 15 meters, these temperatures have been averaged over 5 km segments to reduce small-scale variability that cannot be resolved by the coarser-resolution L4 IST product, following previous approaches^11,18^.

The validation of L4 IST is limited by two primary factors: (1) the increased temporal and spatial sampling errors associated with in situ measurements in ice-covered areas compared to open ocean, and (2) the limited availability of in situ IST observations (Tables 3, 4). The C3S L4 ISTs are generally warmer than the Icebridge observations in the northern hemisphere and colder in the southern hemisphere (Tables 3, 4). These differences likely result from several factors, including spatial and temporal mismatches between the gridded L4 fields (area-averaged over +−24 h) and localized, high-resolution IceBridge transects, as well as limited geographical coverage, diurnal cycle offsets, and potential contamination in IceBridge measurements from low-level clouds in the Arctic.

**Table 3:** Summary of robust validation statistics for the C3S global L4 IST (SIC > 15%) component against reference in situ T2m measurements and skin surface temperature measurements for the northern hemisphere.

Type	MD (^°^C)	RSD (^°^C)	NUM	Period
NP (T2m)	−2.28	3.34	8,106	1982–2012
CRREL (T2m)	−2.76	3.43	23,541	2001–2017
SIMB3 (T2m)	−2.48	3.40	7,591	2019–2024
ECMWF (T2m)	−3.13	3.35	57,611	1993–2014
AWI SB (T2m)	−4.62	2.55	17,615	2013–2024
AWI AWS (T2m)	−3.93	2.55	348	2015–2016
MOSAiC (T2m)	−2.54	2.93	873	2019–2020
MOSAiC (IST)	−2.00	3.49	801	2019–2020
Icebridge (IST)	1.41	3.04	41,351	2012–2019

T2m sources include NP, CRREL, SIMB3, ECMWF, AWI Snow buoys (SB), AWI AWS, and MOSAIC, while skin surface temperatures (IST) are from MOSAIC and Icebridge. Validation metrics include the Median Difference (MD) and Robust Standard Deviation (RSD) of the differences. NUM indicates the number of daily L4 IST matchups with in situ, and Period denotes the time span of available matchups.

**Table 4:** Summary of robust validation statistics for the C3S L4 IST (SIC > 15%) component against reference in situ T2m and skin surface temperature measurements for the southern hemisphere.

Type	MD (^°^C)	RSD (^°^C)	NUM	Period
ECMWF (T2m)	−3.27	3.14	1,912	1999–2009
AWI SB (T2m)	−4.32	3.15	9,051	2013–2024
AWI AWS (T2m)	−1.23	4.99	906	2016–2017
Icebridge (IST)	−2.62	1.99	625	2013

T2m sources include ECMWF, AWI SB and AWI AWS, while skin surface temperatures are from IceBridge. Validation metrics include the Median Difference (MD) and the Robust Standard Deviation (RSD) of the differences. NUM indicates the number of daily L4 IST matchups with in situ, and Period denotes the time span of available matchups.

The validation against MOSAIC IST reveals a median differences of −2.00 ^°^C, indicating that L4 IST is, on average, colder than the corresponding in situ ISTs. The magnitude of this difference varies seasonally, with absolute mean differences below 1 ^°^C from January to April, while peak differences are observed in July and September reaching −4. 8 ^°^C and −3. 4 ^°^C, respectively, for the Met City Station. These results suggest that the L4 IST tends to underestimate surface temperatures in the warmer months. This is likely due to misclassified clouds entering the OI scheme at the time and positions of the MOSAIC observations. In contrast, during winter, the agreement is significantly better, with near-zero or only slightly negative mean difference.

Most in situ observations record T2m, which show median differences from −1.23 ^°^C to −4.62 ^°^C (Tables 3, 4). The largest median differences are obtained from the AWI SBs, reflecting a known warm bias in the snow buoys particularly at low temperatures (summer 1.4 ^°^C, winter: 2.9 ^°^C)^54^. For all in situ sources, the median differences are negative, and part of this can be attributed to real physical temperature gradients between the snow/ice surface and the near-surface atmosphere^30^. The L4 IST validation against MOSAIC T2m shows median differences of −2.54 ^°^C, suggesting an average physical IST–T2m difference of 0.5 ^°^C. This near-surface inversion is a typical feature over sea ice, when the surface is colder than the overlying air due to radiative cooling, which mostly occurs when the absorbed incoming solar radiation is small (during winter and night)^30,55,56^. At the MOSAiC Met City Tower, the gradient is near zero in summer and increases to −0. 5 ^°^C in November-December, and to −1 ^°^C from January to May, consistent with previously reported inversion strengths (−0.42 ^°^C to −2.08 ^°^C with maximum values reaching −3.86^°^ in clear-sky conditions)^30^. These large and temporally varying gradients complicate interpretation of the median differences in Tables 3, 4, and a significant part of the median differences against T2m can likely be attributed to the actual physical skin-T2m differences. The robust standard deviations range from 2.55 ^°^C to 3.49 ^°^C in the Northern Hemisphere, consistent with previous studies^11,18^. In the Southern Hemisphere, RSDs are generally similar, except for the AWI AWS data, which show an elevated value of 4.99 ^°^C.

**Fig. 10:**
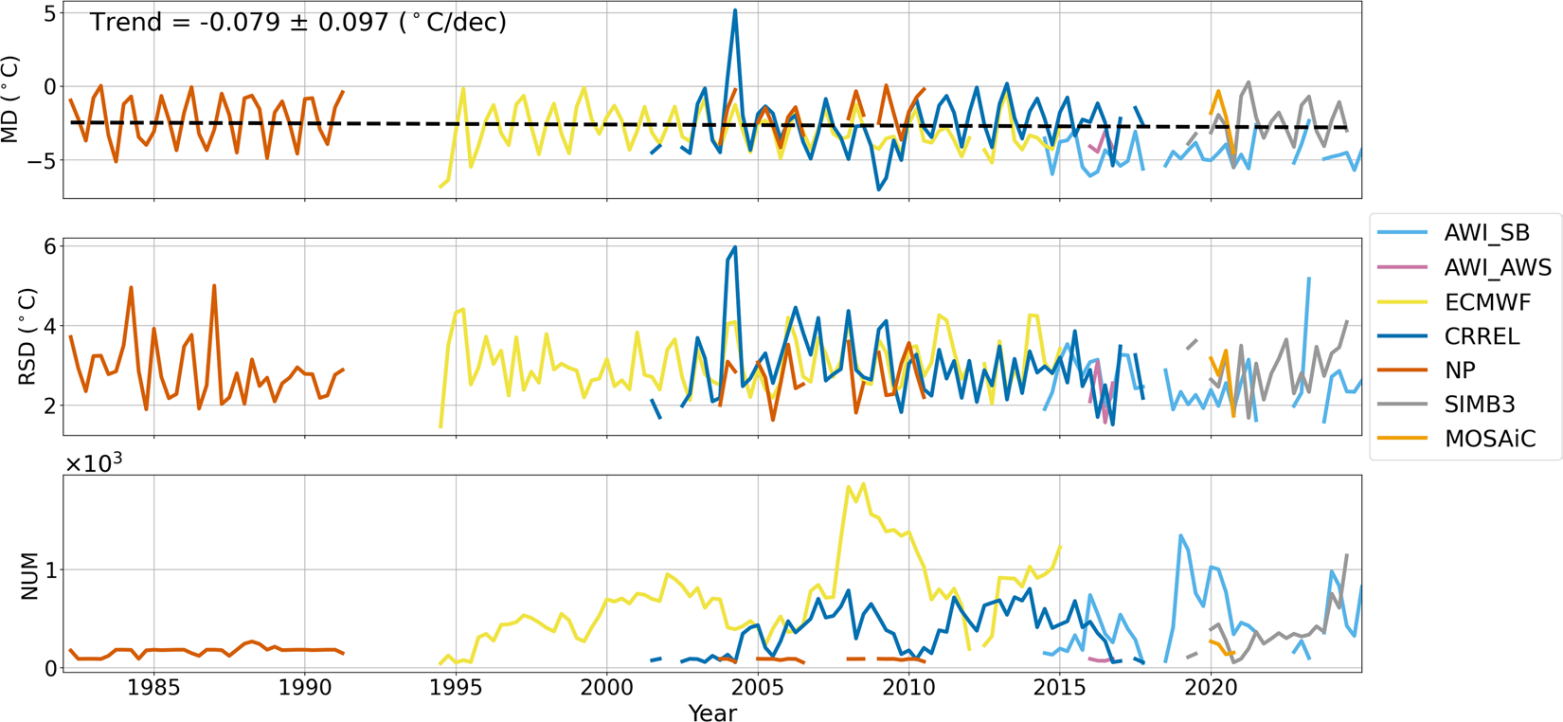
Timeseries of the C3S global L4 IST (SIC > 15%) validation results against in situ observations over sea ice for the Northern Hemisphere in terms of seasonal Median Differences (MD, upper panel), Robust Standard Deviation (RSD, middle panel) and number of matchups (NUM, lower panel). The seasonal median differences, robust standard deviations and trends are only calculated if > 45 matchups are available.

Figure 10 shows the times series of the median differences and robust standard deviations between the L4 IST and in situ T2m for the Northern Hemisphere. Statistics were computed over 3-months periods with at least 45 matchups. The number of matchups, shown in the bottom panel, varies substantially over time. During winter months, the RSDs generally increase, reflecting greater temperature variability and elevated uncertainties in satellite cloud masking during this season^30^. The median differences also exhibit a pronounced seasonal cycle, with larger discrepancies in summer. This pattern is consistent with previous validation results for L3 ISTs^18^ and L4 ISTs^57^. The seasonal pattern is partly attributable to the known and true difference between skin and T2m, while the increased discrepancy during summer aligns with the previous indications of a cold-bias in summer as observed against MOSAiC. The combined (excluding the warm-biased AWI SB) trend in the median temperature difference is −0.08 ^°^C per decade, which is just below the “goal” stability requirement from GCOS of 0.1 ^°^C per decade, supporting the suitability of the C3S L4 SST/IST CDR for long-term climate monitoring. Due to the sparsity of in situ observations, a comparable time series and stability assessment could not be performed for the Southern Hemisphere.

### Uncertainty assessment

**Fig. 11:**
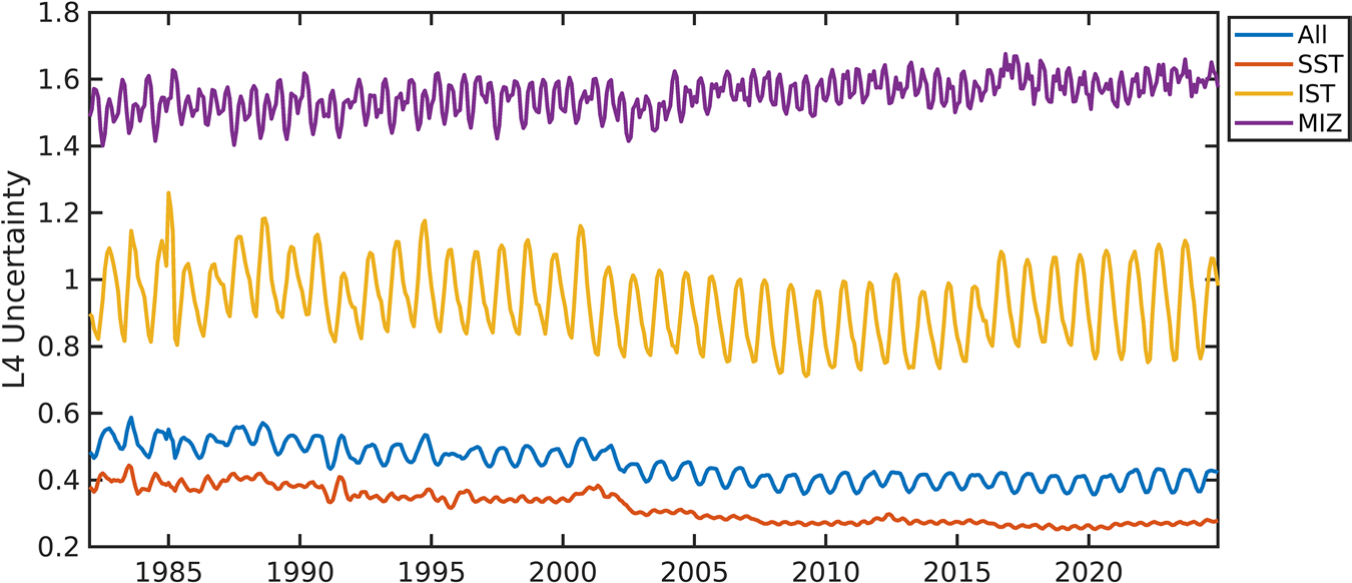
Timeseries of the L4 uncertainty (analysis_error_st) when applying a 3-month moving mean for all surface types combined (All), and separated into uncertainties for SST, IST and the MIZ, using the sea ice fraction.

**Fig. 12:**
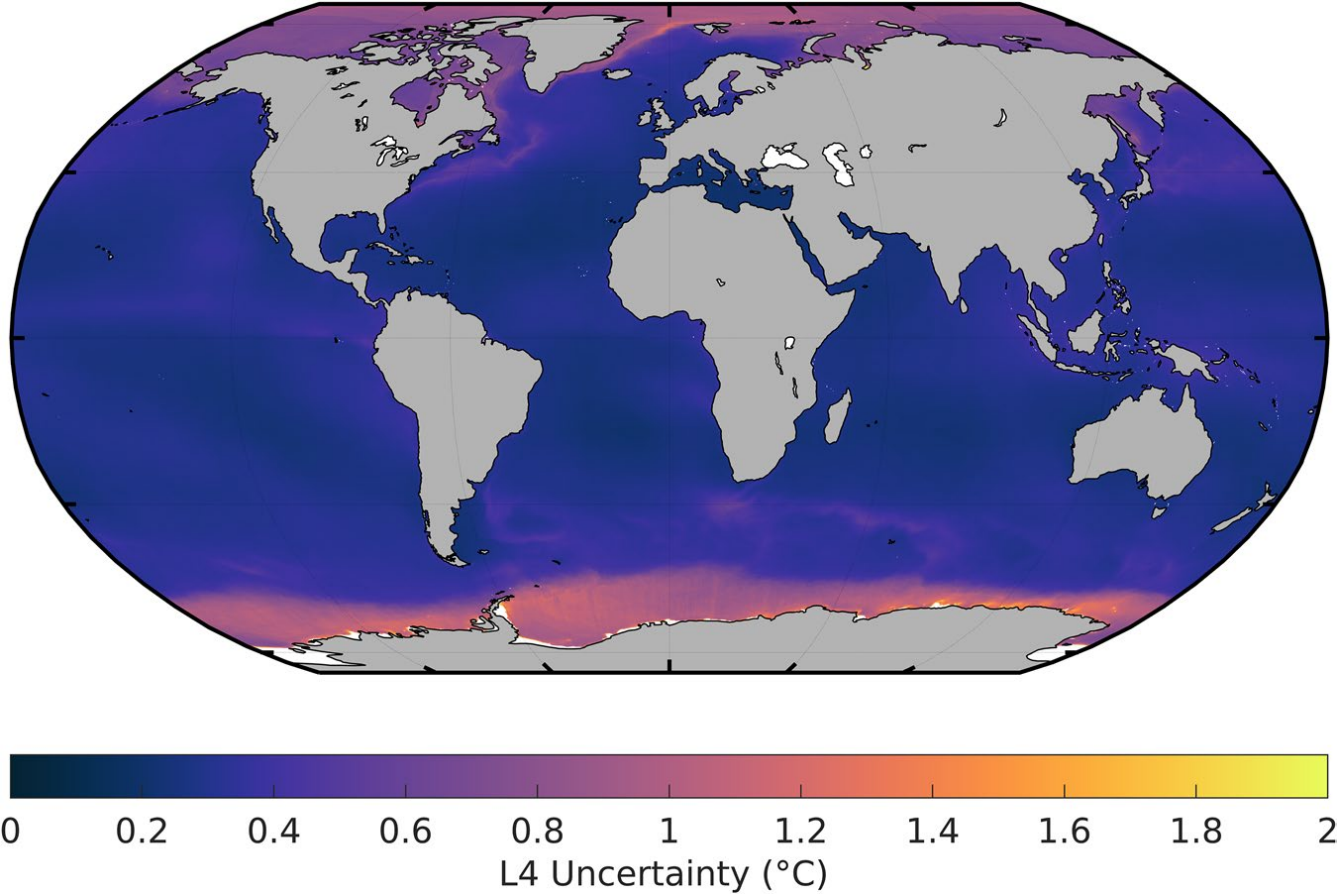
Spatial mean L4 uncertainty (analysis_error_st) over the full record from 1982 to 2024.

The L4 product contains uncertainty estimates of both the SST/UISST (analysed_sst) and the SST/IST (analysed_st) components, with variable names corresponding to these estimates: analysis_error_sst and analysis_error_st, respectively. They are expressed as standard uncertainties (i.e. one standard deviation of the estimated error distribution) and form the focus of this section. The analysis_error_sst consists of two parts: uncertainty attached to the UISST (as explained in the methods section) in sea-ice-covered regions, and uncertainty over open-ocean, which is a direct output from the OI. For the analysis_error_st, the uncertainty estimates are direct outputs from the DMI OI L4 processing scheme and cover both open ocean (SIC < 15%; SST), sea ice (SIC > 70%; IST) and the MIZ (SIC 15–70%). The analysis_error_st is the focus of this section and will be referred to as the L4 uncertainty in the following. Figure 11 shows the time series of the L4 uncertainty estimates for the full record, using a 3-month moving average. The results are shown for all surface types combined, and separately for SST, IST and the MIZ using the sea ice fraction (SIC). When considering the combined uncertainty estimates, there is a general reduction over time, with a noticeable improvement in around 2002, explained by the increasing availability of satellite sensors. This pattern is consistent with the temporal evolution of the SST validation results (Fig. 5). As expected, SST uncertainties are substantially lower than those for sea ice covered regions (IST). This is explained by larger satellite uncertainties, reduced satellite coverage and increased variabilities in sea-ice covered regions. The MIZ uncertainties are higher than those for areas with higher sea ice concentration, mainly explained by a larger first guess variance (reflecting the highly variable nature of the MIZ) and degraded satellite coverage. The times series shown in Fig. 11 represent a mixed signal from both hemispheres. In each case, there is distinct seasonal patterns in the L4 IST and MIZ uncertainties with the largest values occurring during local winter and the smallest during local summer (not shown). This seasonal variability is in good agreement with the IST validation results in Fig. 10, where the largest standard deviations are found during the Northern Hemisphere winter. Figure 12 shows the corresponding spatial mean field of L4 uncertainty (analysis_error_st) over the full record (1982–2024). Consistent with Fig. 11, the largest uncertainties are observed over sea-ice regions, particularly the marginal ice zone. Antarctic sea ice shows higher uncertainties than the Arctic, likely due to both sparser satellite coverage and larger variability in the surface temperatures. Over the open ocean, the largest uncertainties are observed in regions of high variability, such as the Gulf Stream and the Southern Ocean, consistent with the validation results against drifters and Argo (Fig. 7).

**Fig. 13:**
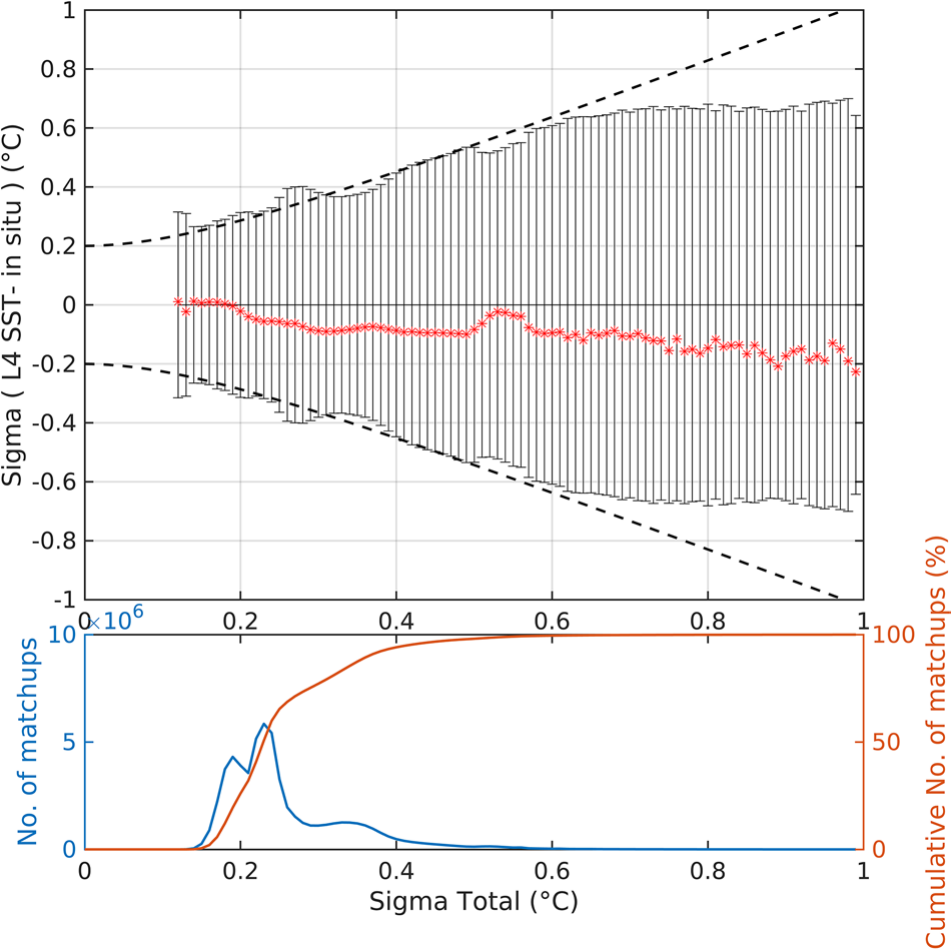
Validation of estimated L4 SST uncertainties against independent in situ observations from drifting buoys. The dashed lines show the theoretical uncertainty when accounting for uncertainties in the drifter SSTs and the sampling error. The solid black lines show one standard deviation of the L4 SST – drifter SST differences for each 0.02 K bin and the red asterisks mark the mean difference. The bottom plots show the number of matchups (blue) and the cumulative percentage of matchups for each bin (red).

The SST uncertainty estimates can be validated by displaying the observed satellite – in situ differences as a function of the estimated uncertainty^16,28,58^ as shown in Fig. 13 using drifters as the reference. Given an estimate of the in situ uncertainty, the expected (theoretical) uncertainty in the satellite – in situ comparison is defined as $\sigma =\sqrt{{\sigma }_{insitu}+{\sigma }_{satellite}}$. The dashed lines in Fig. 13 represent the theoretical uncertainty, assuming that drifters have a total uncertainty of 0.2 K^59^. The red asterisks indicate the observed mean satellite – in situ differences while the bars indicate one standard deviation of the differences within each 0.02 K bin. For uncertainties lower than 0.2 (corresponding to about 50% of the cases), the mean/median differences are very close to zero, while the cases with higher uncertainties tend to be cooler than in situ by about 0.05 to 0.2 K. Overall, the results shows a very good agreement between the estimated and the theoretical uncertainties, particularly in the range between 0.15 and 0.6 K, where the majority of the estimated uncertainties lie. At higher uncertainties, the estimated values tend to be over-estimated. This uncertainty validation approach cannot be applied to the IST component due to the limited availability of high-quality in situ reference observations. As a result, the spatial sampling differences between the L4 IST and in situ near-surface air temperature measurements are dominated by the spatial sampling uncertainty component in the validation analysis.

### Climate assessment

**Fig. 14:**
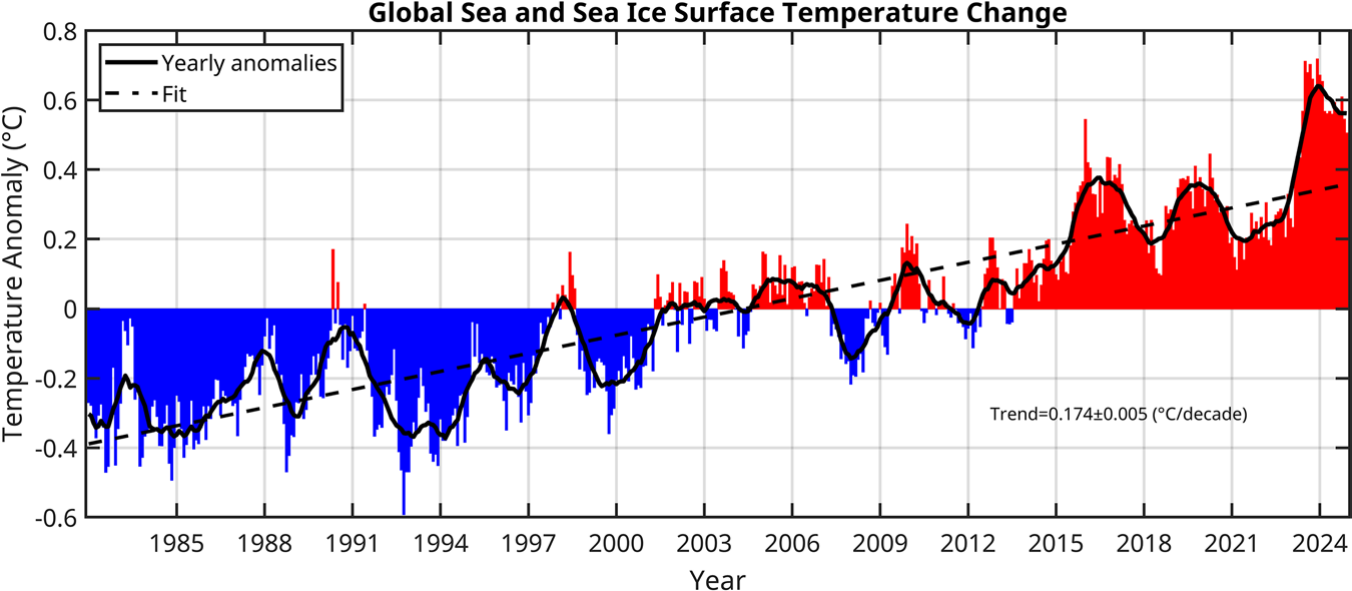
Monthly global anomalies of combined sea and sea-ice surface temperature for 1982–2024, relative to the WMO reference period (1991–2020). Thin colored line show monthly anomaly, the solid black line indicates yearly mean anomalies and the dashed black line represents the linear fit with a slope of 0.174 ^∘^C/decade.

**Fig. 15:**
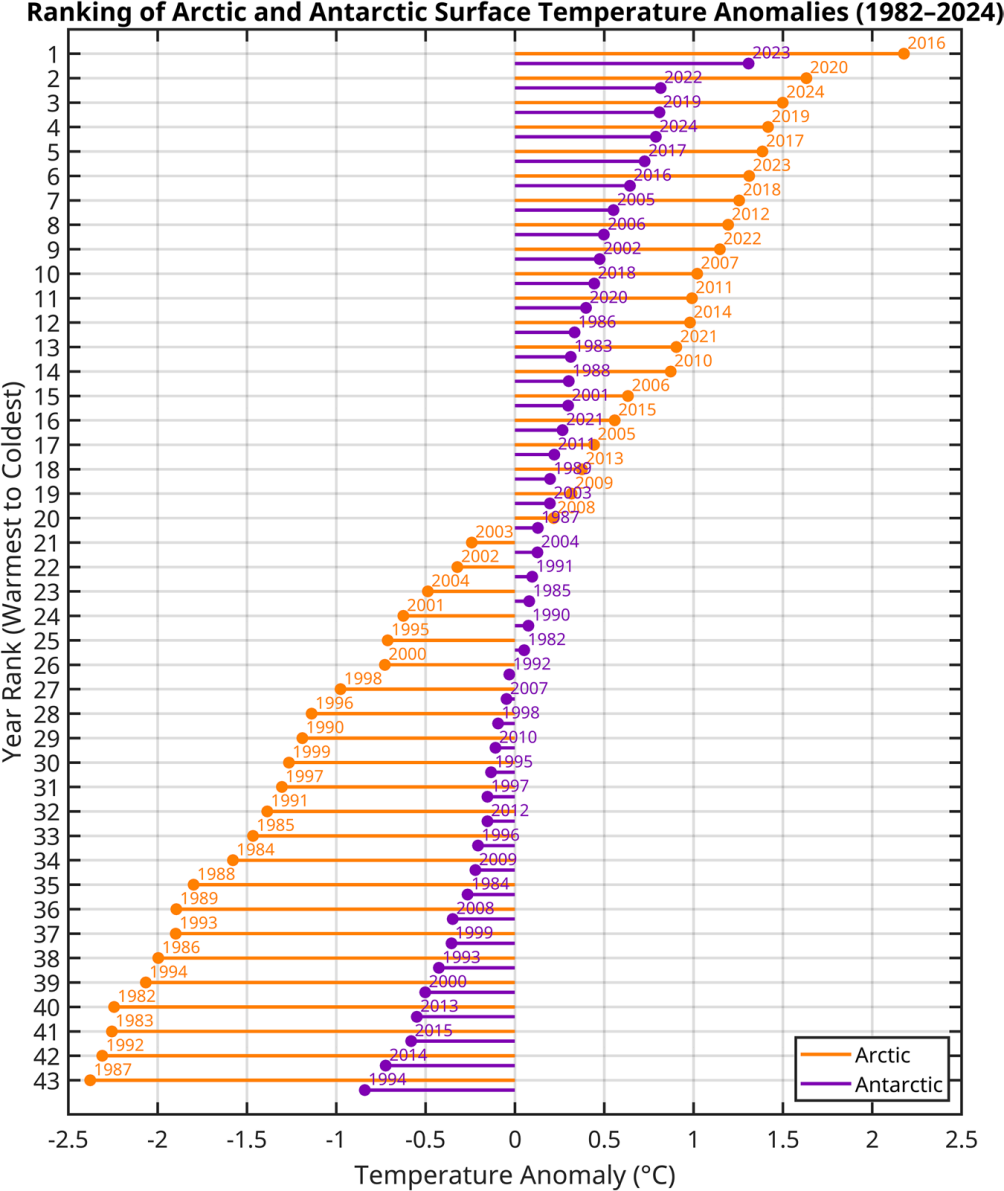
Ranking of the yearly sea and sea-ice surface temperature anomalies for the period 1982–2024 for the Arctic (>60^°^N) and Antarctic (<60^°^S). The anomalies represent the differences between the yearly mean surface temperatures and the average yearly mean surface temperatures of the reference period, 1991–2020, and they are ranked from the warmest to the coldest.

The combination of sea and sea-ice surface temperatures provides a consistent climate indicator, which can be used to monitor day-to-day variations as well as long-term climate trends in the global ocean, including the high latitudes. Figure 14 shows the monthly mean surface temperature anomaly for the period 1982–2024 referenced to the World Meteorological Organization’s (WMO) climatological baseline period (1991–2020). Over the entire period (1982–2024), the fitted linear trend is 0.174 ^°^C per decade, corresponding to an overall increase of approximately 0.75 ^°^C in global combined sea and sea-ice surface temperature over 43 years. The figure also highlights the 2023–2024 exceptionally warm conditions, consistent with recent reports of record high global SST^60,61^. While the global SST/IST trend is largely driven by the ocean surface temperature due to its much larger area compared to sea ice, substantial regional differences exist, particularly in the high-latitude regions. The Arctic (>60^°^N) exhibits a warming trend of 1.014 ^°^C per decade, which is almost six times the global average warming trend, resulting in total increase ~4.36 ^°^C from 1982–2024. In contrast, the Antarctic region (<60^°^S) shows a weaker warming trend of 0.125 ^°^C per decade resulting in a total surface temperature increase of ~0.54 ^°^C for the data record. Despite the relatively weak long-term trend in the Antarctic region there has been a recent emergence of unusually warm years. To illustrate this, we examine the annual rankings of surface temperature anomalies for both the Arctic and Antarctic region (Fig. 15). The annual anomalies are ranked from warmest to coldest to highlight the frequency and distribution of extreme surface temperature years. The Arctic region shows a clear and persistent warming signal, with the ten warmest years all occurring since 2007. This pronounced Arctic warming is consistent with previous findings^11,62,63^ and is attributed to the large reduction in sea ice cover^64^ and several positive feedback mechanisms operating in the region^65–67^. Conversely, the Antarctic rankings reveal large inter-annual variability and a much weaker long-term warming trend of the surface. The weaker warming and higher variability are consistent with previous studies and are largely attributed to the Southern Ocean’s meridional overturning circulation, together with its substantial heat uptake and thermal inertia, which redistribute excess heat into deeper layers rather than the surface^68,69^. In addition, strong circumpolar winds and the Antarctic Circumpolar Current act to dynamically isolate the region, limiting meridional heat transport and contributing to regional variability^70^. The presence of extensive sea-ice cover further modulates the sea and sea-ice surface temperatures and contributes to enhanced year-to-year variability, as Antarctic sea ice exhibits large inter-annual fluctuations^71,72^. The asymmetry between the two poles is evident in the distribution of ranked years, with many of the coldest years still occurring in the latter half of the record for the Antarctic, whereas recent Arctic years are consistently among the warmest. However, the ranking of annual Antarctic surface temperature anomalies also reveals a notable shift in recent years with several of the warmest years occurring within the last decade, particularly between 2016 and 2024. This clustering of warm years contrasts with earlier decades, where warm and cold years appeared more randomly distributed, and is consistent with the recent period of exceptionally low Antarctic sea-ice extent^12^. The recent sequence of anomalously warm years may indicate that the Antarctic surface temperature response is beginning to deviate from its previously stable or weakly warming trajectory, which highlights the importance of continued and consistent monitoring of this region.

## Usage Notes

### Sparse observations in early years

In the 1980s, fewer input observations are available^16^. This is challenging for the OI scheme and resulting in increased uncertainty in the L4 SST/IST fields. This is evident from the larger standard deviations against in situ SST observations in the earlier years (Fig. 5). However, the increased uncertainty is reflected in elevated values of the provided uncertainty fields (analysis_error_st and analysis_error_sst) and is clearly shown in Fig. 11, where higher uncertainties are observed for both SST and IST during the early part of the record.

### Diurnal sampling and clear-sky bias

For sea ice, the dataset is derived solely from satellite observations under clear-sky conditions, which may introduce sampling biases. However, the diurnal variability in IST can be substantial, especially during periods of strong solar forcing, but this is not fully resolved as the L2P input observations are not uniformly distributed across the diurnal cycle. In addition, clear-sky conditions can lead to systematic differences relative to all-sky averages, with a tendency toward a cold bias over sea ice due to enhanced radiative cooling in the absence of cloud cover. This effect is partially mitigated through the clear-sky bias correction added to the AASTI/C3S L3 IST fields, based on previous findings^30^. However, users should note that this correction is assumed to be constant, which is unlikely to fully capture variability associated with changes in diurnal sampling and seasonal conditions.

### Skin and depth temperature

The dataset combines skin surface temperature (IST) over sea ice with sea surface temperatures (SST) representative of approximately 20 cm depth in open ocean. Users should be aware of this difference, in particular when analysing transitions between ice-covered and open-water regions.

### Limited in situ validation over sea ice

Validation of the dataset is constrained by the scarcity of in situ observations over sea ice, particularly in the Southern Hemisphere. There is a clear need for additional dedicated in situ measurements of ice surface (skin) temperature, similar to the high-quality fiducial reference measurements (FRMs) collected during the MOSAiC campaign. Such FRMs are essential to enable robust validation, improve the accuracy of surface temperature estimates, and provide a better understanding of the differences between skin temperature and near-surface air temperature over sea ice. The importance of high-quality FRMs has previously been highlighted as a key observational need, particularly in sea ice regions and the marginal ice zone^73^.

### Performance in the Marginal Ice Zone

Surface temperature estimates in the marginal ice zone (MIZ) are subject to increased uncertainty due to limited satellite coverage, sampling limitations, mixed surface conditions, elevated uncertainties in the sea ice concentration (SIC) field, and challenges in the optimally interpolating observations originating from both ice and open water. The current approach relies on accurate SIC information to ensure that the appropriate surface temperature observations (SST, IST, or MIZT) are used. Any errors in SIC can thereby lead to misclassification of surface type and the inappropriate inclusion or exclusion of observations. Further improvements could be achieved by a daily, high-resolution SIC products (e.g. SAR-based datasets such as ASIP^74^. Such information would support a more accurate separation between SST and IST retrieval algorithms, and enable observation weighting within the OI scheme to reflect the actual SIC value. Although post-processing steps have been applied to improve consistency between surface temperature and SIC fields, it remains difficult to determine which dataset is more accurate, particularly considering mismatches in the temporal and spatial scales of the SST/IST and SIC observations. None of the in situ validation points currently cover the MIZ, and observational data in this region are generally very limited. This restricts robust accuracy assessment and highlights the need for dedicated measurement campaigns to improve validation, enhance understanding of surface temperature characteristics, and refine the SIC field in this complex region. The uncertainty estimates provided with the dataset are correspondingly elevated in the MIZ, and users are advised to take these into account when using the data.

### Potential summer cold bias over Arctic sea ice

A cold bias was observed during the summer of the MOSAiC validation campaign. Since MOSAiC represents only a single year, it is unclear whether this bias is specific to that period and location (e.g., due to misclassification of clouds as clear sky) or reflects a more general issue, potentially related to sparse satellite observations during summer, retrieval limitations, diurnal sampling, or cloud contamination. Additional in situ observations are needed to resolve this uncertainty.

## Data Availability

The daily sea surface and sea-ice temperature data used in this study are available from the Copernicus Climate Change Service (C3S) Climate Data Store (CDS)^40^ and distributed under the Creative Commons Attribution 4.0 International license (CC-BY 4.0; https://creativecommons.org/licenses/by/4.0/). Regular monthly updates are planned within C3S. Neither the European Commission nor ECMWF is responsible for any use that may be made of the Copernicus information or data it contains.

## Code availability

The codes used to validate the sea surface temperatures and to conduct the climate assessment are available here: https://github.com/piaenglyst/global_sst_ist/tree/main.
